# Construction and validation of a risk prediction model for 3- and 5-year new-onset atrial fibrillation in HFpEF patients

**DOI:** 10.3389/fcvm.2024.1429431

**Published:** 2024-08-16

**Authors:** Shuaishuai Wang, Zhonglei Xie, Fengjiao Wang, Wenzhong Zhang

**Affiliations:** ^1^ Department of Cardiology, Affiliated Hospital of Qingdao University, Shandong, China; ^2^ Institutes of Biomedical Sciences, Fudan University, Shanghai, China

**Keywords:** new-onset atrial fibrillation (NOAF), HFpEF, risk prediction model, echocardiography, nomogram

## Abstract

**Background:**

Patients with heart failure (HF) with preserved ejection fraction (HFpEF) are more prone to atrial fibrillation (AF) compared to those with heart failure with reduced ejection fraction (HFrEF). Nevertheless, a risk prediction model for new-onset atrial fibrillation (NOAF) in HFpEF patients remains a notable gap, especially with respect to imaging indicators.

**Methods:**

We retrospectively analyzed 402 HFpEF subjects reviewed at the Affiliated Hospital of Qingdao University from 2017 to 2023. Cox regression analysis was performed to screen predictors of NOAF. A nomogram was constructed based on these factors and internally validated through the bootstrap resampling method. A performance comparison between the nomogram and the mC_2_HEST score was performed.

**Results:**

Out of the 402 participants, 62 (15%) developed atrial fibrillation. The risk factors for NOAF were finally screened out to include age, chronic obstructive pulmonary disease (COPD), hyperthyroidism, renal dysfunction, left atrial anterior–posterior diameter (LAD), and pulmonary artery systolic pressure (PASP), all of which were identified to create the nomogram. We calculated the bootstrap-corrected C-index (0.819, 95% CI: 0.762–0.870) and drew receiver operator characteristic (ROC) curves [3-year areas under curves (AUC) = 0.827, 5-year AUC = 0.825], calibration curves, and clinical decision curves to evaluate the discrimination, calibration, and clinical adaptability of the six-factor nomogram. Based on two cutoff values calculated by X-tile software, the moderate- and high-risk groups had more NOAF cases than the low-risk group (*P* < 0.0001). Our nomogram showed better 3- and 5-year NOAF predictive performance than the mC_2_HEST score estimated by the Integrated Discriminant Improvement Index (IDI) and the Net Reclassification Index (NRI) (*P* < 0.05).

**Conclusions:**

The nomogram combining clinical features with echocardiographic indices helps predict NOAF among HFpEF patients.

## 1 Introduction

Heart failure (HF) and atrial fibrillation (AF) share common risk factors and pathophysiologic mechanisms, so they often coexist like twins (1), leading to poor prognosis together, and their co-occurrence indicates a worse outcome than either one alone. Studies have proven that HFpEF patients are more likely to develop AF than HFrEF at any time (62% vs. 55%, *P* = 0.02) (2). In the sequence of HFpEF and AF, the later onset of AF in patients with HFpEF tends to be associated with a worse prognosis (2, 3). If atrial fibrillation is recognized early, therapeutic anticoagulation can reduce the absolute risk of all strokes by 2.7% per year for primary prevention and 0.5% for mortality (4). Thus, the guidelines of numerous professional societies suggest screening for AF proactively (5, 6). The risk prediction model is crucial for disease screening and clinical practice. It accurately and efficiently distinguishes low- and high-risk populations, enabling a rational allocation of screening strategies. The nomogram and risk score involved in this study are visual and practical ways of presenting risk prediction models conducive to clinical application.

Several predictive models have been recommended to assess the risk of new-onset atrial fibrillation (NOAF) in asymptomatic people (7–13), such as the Atherosclerosis Risk in Communities Study (ARIC) score, the Framingham Heart Study (FHS) score, and the Cohorts for Heart and Aging Research in Genomic Epidemiology—Atrial Fibrillation (CHARGE-AF) score. However, most of these are designed based on Western community-based populations, which greatly limits the generalized application of these risk scores in Asian populations, especially in hospital-based Chinese populations. In addition, some of the laboratory variables in these scores are not easily available (10–13). For this reason, Li et al. (14) created a simple clinical tool using the Yunnan Insurance Database of China, the C_2_HEST score, to assess the hazard of NOAF in individuals without structural heart disease (SHD). Later, this risk score was further stratified by age to develop the mC_2_HEST score (age ≥75 years old and systolic heart failure score, 2 points each; age 65–74 years old, coronary heart disease, hypertension, chronic obstructive pulmonary disease (COPD), and hyperthyroidism score, 1 point each) in the hospital population (15, 16). However, a growing number of studies have found that HF with diastolic dysfunction is more closely related to AF and has a poorer prognosis than systolic heart failure (17, 18). Additionally, echocardiography has been considered an important diagnostic and prognostic tool for the management of patients with arrhythmias, especially in the field of atrial fibrillation and ventricular arrhythmias (19). Studies have found a correlation between echocardiography and both maintenance of sinus rhythm success rate and thromboembolism risk (20). Montserrat et al. (21) showed that the left atrial size by echocardiography is related to the success of radiofrequency catheter ablation for atrial fibrillation. Sánchez et al. (22) demonstrated through preoperative echocardiographic analysis that atrial volume and end-diastolic pressures are sensitive markers of postoperative atrial fibrillation. The mC_2_HEST score was constructed using only basic clinical information and did not include any imaging indicators. It is yet to be explored whether the inclusion of echocardiography provides added value to the assessment based only on clinical factors.

Therefore, our study aims to establish a more suitable NOAF risk prediction model for hospital-oriented HFpEF patients by using the data platform in the Affiliated Hospital of Qingdao University. Moreover, the model will be plotted into a nomogram to provide clues for high-risk AF patients.

## 2 Methods

### 2.1 Study population

The Affiliated Hospital of Qingdao University has an electronic medical record database, called “Yidu Yun,” established in 2012. This database assigned a permanent personal registration number to each patient that recorded their medical history, including diagnosis, treatment, bodily examination, imaging data, and laboratory results. Each hospitalization was classified according to the ICD-10 or ICD-9. The studied subjects were limited to HFpEF patients receiving regular outpatient care from 1 January 2017 to 1 October 2023. This study was limited to adults (>18 years of age). The calculation of the mC_2_HEST and CHA_2_DS_2_-VASc scores was in accordance with the respective original descriptions. The following subjects were excluded: (1) patients diagnosed with atrial fibrillation before being included in this study; (2) implanted with cardiac electronic devices, such as pacemakers, implantable cardioverter-defibrillators, or resynchronization devices of the heart before inclusion in the study; (3) with valvular heart diseases, such as rheumatology with severe valvular disease or artificial heart valve; (4) with cardiomyopathy; (5) with advanced disease and limited life expectancy or with metastatic cancer and moderate or more severe dementia; (6) with critical data missing; and (7) with number of reviews ≤2/year. The sample size was calculated using the clinical prediction model EPV empirical guidelines. The results are stable and valid when the population of positive events is 5–10 times the number of variables ultimately screened (23). Studies have confirmed that the prevalence of atrial fibrillation rises with the severity of heart failure, ranging from 5% of NYHA I, asymptomatic HF, to 50% of NYHA IV (24). Research on the correlation between AF and HF over time has revealed that over one-third of HF patients experience AF later in life. Among these patients, 18% were diagnosed with AF within 30 days of their heart failure diagnosis, while 12% developed AF after a more extended period after HF diagnosis (2). We calculated and added 10% invalid samples based on the 12% incidence rate: the sample size was 278–556. Finally, this study included 402 patients in total.

### 2.2 Candidate predictors

Based on previous reports and convenient clinical access principle, the following candidate predictors were enrolled: demographic characteristics and basic clinical information for age, sex, body mass index (BMI), smoking and drinking status, hypertension, diabetes, coronary artery disease (CAD), acute myocardial infarction (AMI), history of percutaneous coronary intervention or bypass surgery (PCI/CABG), peripheral vascular disease (PVD), chronic obstructive pulmonary disease (COPD), ischemic stroke, hyperthyroidism, hypothyroidism, anemia, and renal dysfunction. For echocardiographic indices, we selected the left atrial anterior–posterior diameter (LAD), the ratio of the interventricular septum (IVS) and left ventricular posterior wall (LVPW), and pulmonary artery systolic pressure (PASP). Calculate risk score, including the CHA_2_DS_2_-VASc score (congestive HF, hypertension, diabetes, age 65–74, vascular disease, and female score, 1 point each; ischemic stroke and age ≥75 score, 2 points each) and mC_2_HEST score. In the current study, we did not assign any point to the “systolic heart failure” category in the mC_2_HEST score because the patients we included were HFpEF patients with only mild abnormalities in systolic function.

### 2.3 Statistical analysis

In the baseline characteristics of enrolled subjects, categorical variables were described as percentages or frequencies (%), while continuous variables were described as mean ± SD or median and interquartile spacing. For model construction, candidate predictors for NOAF were first identified through univariate Cox regression analysis. Variables with *P* < 0.1 and definite clinical significance would be included in the multivariate Cox regression analysis. The minimum AIC by stepwise backward regression was used to determine the final predictors and construct a nomogram. All interior validations were carried out with the use of the bootstrapping approach with 500 resamples. Statistical analysis was performed using R software (ver. 4.2.3). The nomogram and calibration curves were plotted by the rms package in R. The receiver operator characteristic (ROC) curves were plotted by the survival ROC package. The decision curve analysis (DCA) curves were plotted by the survival package. The time-dependent C-index curves were plotted via the pec package. The X-tile software was used to determine the cutoff values of the low-, medium-, and high-risk nomograms predicting the AF events in HFpEF patients. The survival curve was plotted using Kaplan–Meier survival analysis and compared using the log-rank test with the survminer and ggplots packages. The models were compared using the nricens package and the SURVIDINRI package to calculate the Net Reclassification Index (NRI) and the Integrated Discriminant Improvement Index (IDI).

### 2.4 Definition of HFpEF and NOAF

According to the 2021 ESC Guidelines, the definition of HFpEF should include the following criteria (25): (1) symptoms and signs of HF, such as breathlessness, orthopnea, ankle swelling, and third heart sound (gallop rhythm); (2) a left ventricular ejection fraction (EF) of ≥50%, which is mainly confirmed by transthoracic echocardiography; and (3) objective evidence of cardiac structural and/or functional abnormalities consistent with the presence of the LV diastolic dysfunction/raised LV filling pressures, including E/e′ > 9, NT-proBNP > 125 pg/ml, or BNP > 35 pg/ml (sinus rhythm). NOAF following HFpEF was the outcome variable in this study mainly defined by the ECG or 24-h Holter results. The beginning of atrial fibrillation-related treatment, including anticoagulants, antiarrhythmic drugs, cardioversion, and ablation, also indicated the occurrence of AF. In addition, atrial flutter was also included. Although the two conditions were different in electrophysiology, most atrial flutter sufferers have developed or are about to develop atrial fibrillation. Even the risk of stroke is similar between them (13, 26).

### 2.5 Ethics and study quality control

The study adhered to the TRIPOD statement and fulfilled all the guidelines' requirements. The Ethics Review Committee of Qingdao University approved the study. Informed consent was waived as the project anonymized patient data through a retrospective research method.

## 3 Results

### 3.1 Patient characteristics

**Figure 1:**
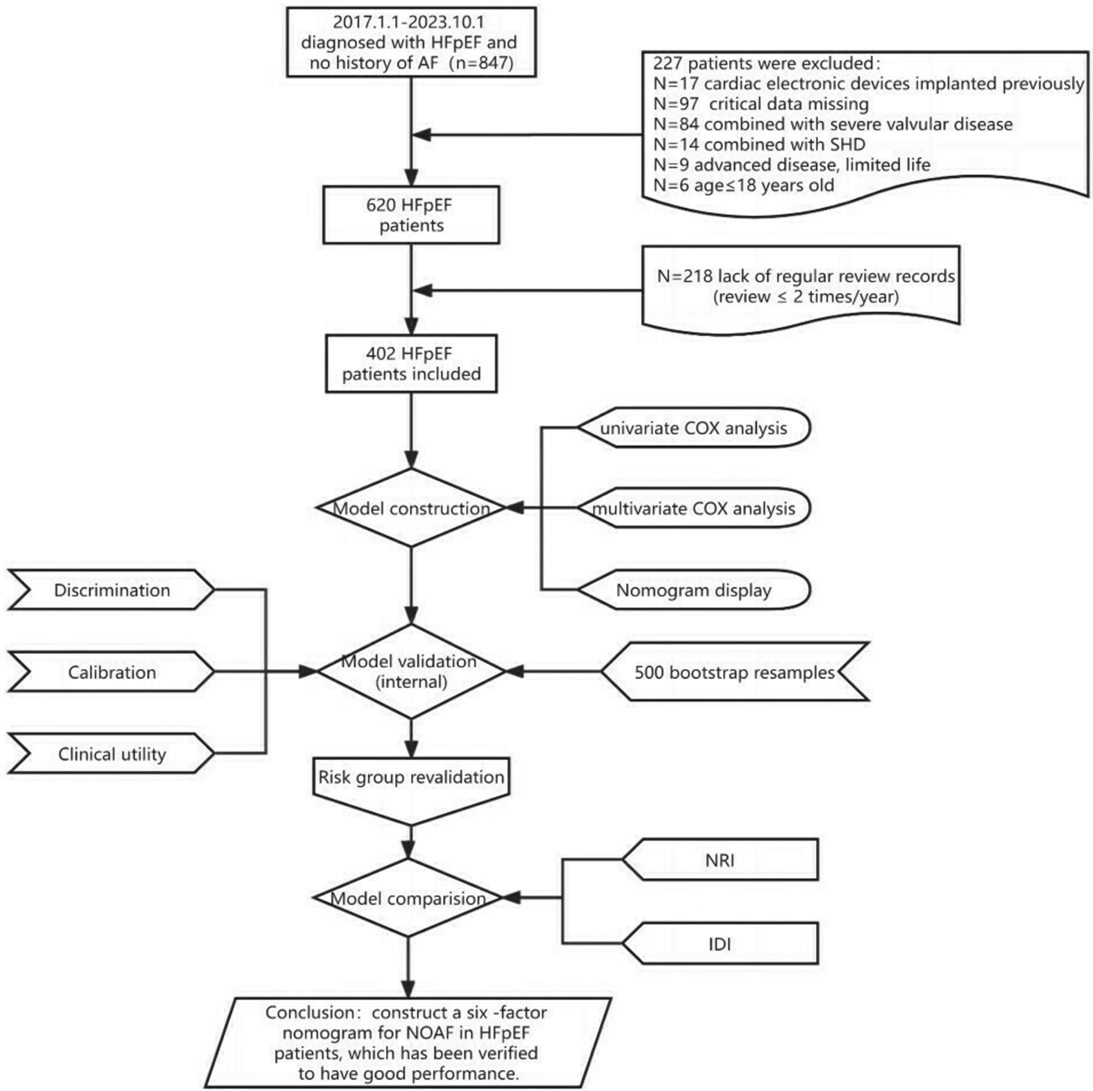
Flow diagram of the study design.

As shown in the flowchart presented in Figure 1, we recruited 402 HFpEF subjects with complete baseline clinical and echocardiographic dates, and 62 patients developed AF within a nearly 7-year follow-up period, with an average follow-up time of 53.08 months. Their baseline characteristics were reported in Table 1, which described the HFpEF patients with NOAF and without NOAF. We set different strategies for the “Age” variable, including the quantitative variable “Age” and the classification variable “Age2” in the statistical analysis. Finally, we calculated the CHA_2_DS_2_-VASc score and mC_2_HEST score for the NOAF patients as 4.90 ± 1.64 points and 2.69 ± 1.11 points, respectively. Subjects without NOAF scored 4.01 ± 1.69 and 1.96 ± 0.98, respectively.

**Table 1:** Baseline characteristics of 402 subjects.

Characteristics	Non-NOAF (*n* = 340)	NOAF (*n* = 62)
Age, mean ± SD (years)	62.61 ± 9.68	69.82 ± 10.75
Age2, *n* (%)		
<65	196 (57.65)	15 (24.19)
65–74	104 (30.59)	29 (46.77)
≥75	40 (11.76)	18 (29.03)
Female sex, *n* (%)	149 (43.82)	28 (45.16)
BMI, mean ± SD (kg/m^2^)	25.69 ± 3.57	26.06 ± 3.58
Drink, *n* (%)	81 (23.82)	16 (25.81)
Smoke, *n* (%)	106 (31.18)	18 (29.03)
Hypertension, *n* (%)	253 (74.41)	52 (83.87)
Diabetes, *n* (%)	131 (38.53)	26 (41.94)
CAD, *n* (%)	216 (63.53)	33 (53.23)
AMI, *n* (%)	130 (38.24)	20 (32.26)
PCI/CABG, *n* (%)	196 (57.65)	17 (27.42)
PVD, *n* (%)	42 (12.35)	16 (25.81)
COPD, *n* (%)	8 (2.35)	9 (14.52)
Ischemic stroke, *n* (%)	74 (21.76)	21 (33.87)
Hyperthyroidism, *n* (%)	4 (1.18)	8 (12.90)
Hypothyroidism, *n* (%)	19 (5.59)	1 (1.61)
Anemia, *n* (%)	26 (7.65)	14 (22.58)
Renal dysfunction, *n* (%)	20 (5.88)	22 (35.48)
Hyperlipidemia, *n* (%)	117 (34.41)	22 (35.48)
IVS/LVPW ratio	1.09 ± 0.12	1.09 ± 0.12
LAD, mean ± SD (cm)	3.67 ± 0.36	3.96 ± 0.48
PASP, mean ± SD (mmHg)	29.84 ± 7.19	38.34 ± 13.56
CHA2DS2-VASc	4.01 ± 1.69	4.90 ± 1.64
mC2HEST	1.96 ± 0.98	2.69 ± 1.11

NOAF, new-onset atrial fibrillation; CAD, coronary artery disease; AMI, acute myocardial infarction; PCI, percutaneous coronary intervention; CABG, coronary aorta bypass grafting; PVD, peripheral vascular disease; COPD, chronic obstructive pulmonary disease; IVS/LVPW, interventricular septum/left ventricular posterior wall; LAD, left atrial anterior–posterior diameter; PASP, pulmonary artery systolic pressure.

### 3.2 Construction of the nomogram based on clinical and echocardiographic data

**Figure 2:**
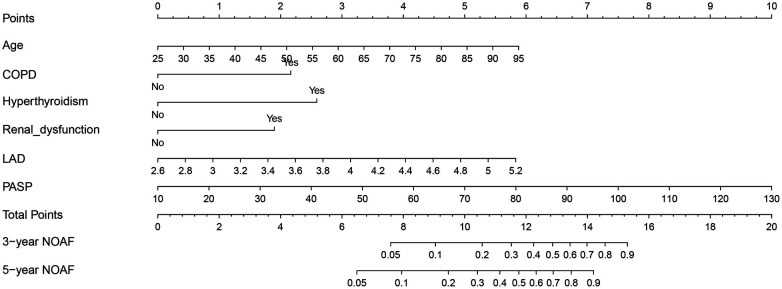
Nomogram based on age, PVD, COPD, hyperthyroidism, renal dysfunction, LAD, and PASP in prediction for 3- and 5-year NOAF of HFpEF patients.

Table 2 presents the detailed outcomes of both univariate and multivariate analyses. Based on univariate Cox regression, Age, Age2, PCI/CABG, PVD, COPD, hyperthyroidism, anemia, renal dysfunction, LAD, and PASP were found to be associated with NOAF in patients with HFpEF (*P* < 0.1). Considering that “Age” and “Age2” are essentially the same, they have a high correlation. We found that survival time was no statistical difference among the three groups of Age2 by the K–M method (log-rank *P* = 0.107), so we chose the continuity variable “Age” instead of the classification variable “Age2” in the multivariate analysis. In addition, in the univariate analysis of the effect of PCI/CABG on NOAF, it seemed unreasonable that PCI/CABG was the protective factor of NOAF (HR = 0.30, 95% CI:0.17–0.52). The reason can be found in the baseline table (Figure 1). The proportion of patients without atrial fibrillation who had PCI/CABG surgery history was significantly higher than the NOAF group (57.65% vs. 27.42%), and the CAD history was also higher than the NOAF group (63.53% vs. 53.23%). It is possible that timely PCI/ CABG surgery can avoid the occurrence of late AF in HFpEF patients with CAD, which does not represent all subjects, so we also chose to give up the variable “PCI/CABG.” We further studied the non-NOAF and NOAF groups of patients with CAD. We found that the most common clinical presentation of CAD was non-ST-segment elevation myocardial infarction–unstable angina (NSTEMI-UA) in both groups (80.56% and 90.91%). The proportion of ST-segment elevation myocardial infarction (STEMI) in the non-NOAF group was higher than that in the NOAF group (19.44% vs. 9.09%), and the proportion of NSTEMI-UA was lower (80.56% vs. 90.91%). The remaining variables were analyzed by the stepwise backward method in multivariate Cox regression analysis. Finalized age (HR = 1.04, 95% CI: 1.01–1.07; *P* = 0.003), COPD (HR = 2.91, 95% CI: 1.26–6.71; *P* = 0.012), hyperthyroidism (HR = 3.59, 95% CI: 1.59–8.10; *P* = 0.002), renal dysfunction (HR = 2.55, 95% CI: 1.43–4.56; *P* = 0.002), LAD (HR = 3.02, 95% CI: 1.57–5.82; *P* = 0.001), and PASP (HR = 1.04, 95% CI: 1.03–1.06; *P* < 0.001) were the six factors identified as the NOAF independent predictors (Table 2). Based on the above results, we constructed a nomogram using these six predictors and named it the “APART” nomogram (Figure 2). The first “A” represents age; “P” stands for pulmonary diseases, including COPD and PASP; the second “A” signifies the anteroposterior diameter of the left atrium; “R” represents renal dysfunction; and “T” stands for hyperthyroidism. Summing the scores for all variables on the “Points” axis produces the corresponding predictions.

**Table 2:** Univariate and multivariate Cox proportional hazard analyses of HFpEF with new-onset AF patients.

Risk factors	Univariate analysis			Multivariate analysis		
	HR	95% CI	*P*	HR	95% CI	*P*
Age	1.08	1.05–1.11	<.001	1.04	1.01–1.07	0.003
Age2						
<65						
65–74	3.27	1.75–2.61	<0.001			
≥75	5.17	6.10–10.26	<0.001			
Female sex	1.10	0.67–1.81	0.714			
BMI	1.02	0.96–1.10	0.512			
Drink	0.97	0.55–1.71	0.912			
Smoke	0.79	0.46–1.38	0.413			
Hypertension	1.30	0.66–2.55	0.455			
Diabetes	1.09	0.66–1.81	0.728			
CAD	0.68	0.41–1.12	0.132			
AMI	0.79	0.46–1.35	0.390			
PCI/CABG	0.30	0.17–0.52	<0.001			
PVD	2.08	1.18–3.67	0.012			
COPD	5.37	2.64–10.91	<0.001	2.91	1.26–6.71	0.012
Ischemic stroke	1.55	0.91–2.61	0.105			
Hyperthyroidism	5.85	2.77–12.35	<0.001	3.59	1.59–8.10	0.002
Hypothyroidism	0.29	0.04–2.07	0.215			
Anemia	2.81	1.55–5.09	<0.001			
Renal dysfunction	5.25	3.12–8.84	<0.001	2.55	1.43–4.56	0.002
Hyperlipidemia	0.94	0.56–1.58	0.819			
IVS/LVPW ratio	1.26	0.14–11.31	0.837			
LAD	6.49	3.31–12.74	<0.001	3.02	1.57–5.82	0.001
PASP	1.04	1.03–1.06	<0.001	1.04	1.03–1.06	<0.001

### 3.3 Discrimination, calibration, and clinical utility

The performance of the “APART” nomogram was assessed through discrimination, calibration, and clinical utility. We used 500 bootstrap resampling methods to verify internally.

**Figure 3:**
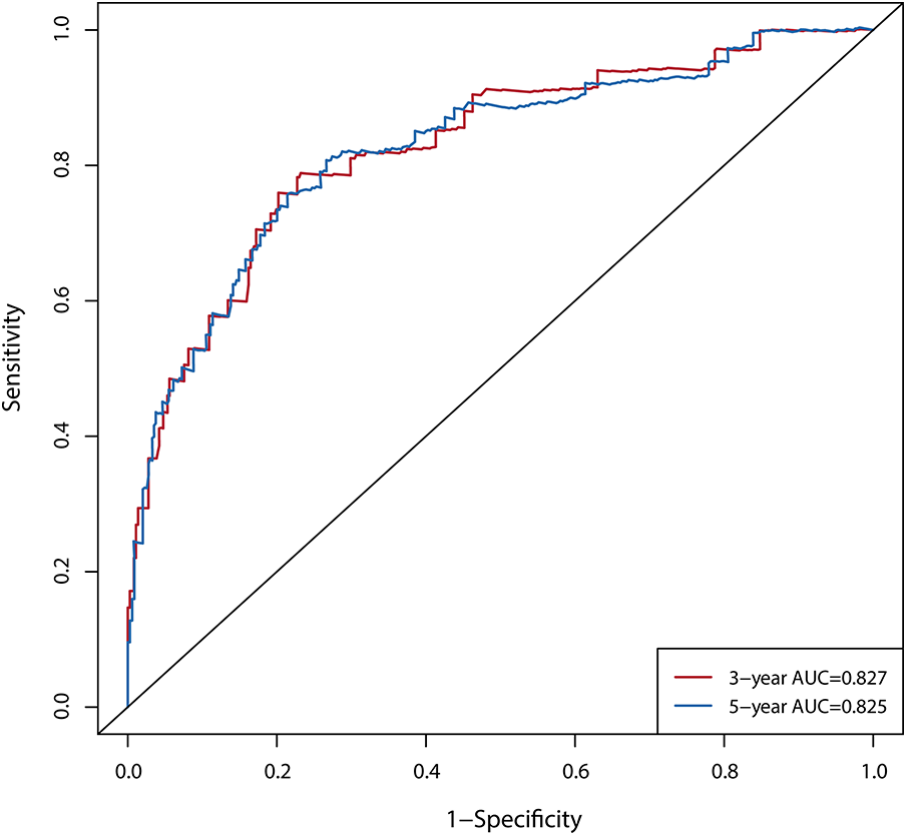
ROC curves of the 3- and 5-year nomogram for NOAF of HFpEF patients.

**Figure 4:**
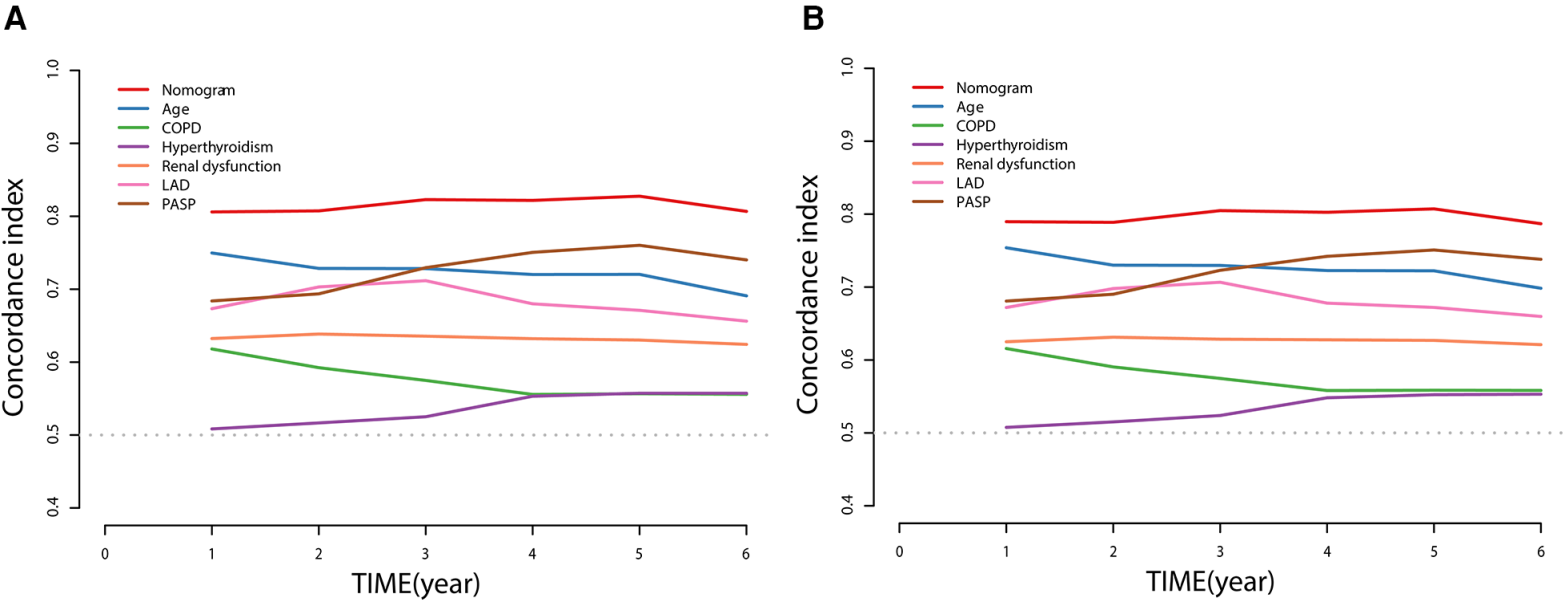
Time-dependent C-index of nomogram compared with any single prognostic marker for NOAF of HFpEF patients **(A)** and internally validated using a bootstrap resampling method **(B)**.

Discriminative power was assessed using the receiver operator characteristic (ROC) curves, areas under curves (AUC), and C-index curves. The AUC of the nomogram was 0.827 and 0.825 across the 3- and 5-year predictions, respectively (Figure 3). The time-dependent C-index curves further confirmed that our established nomogram's discriminative accuracy consistently outperformed any predictor alone (Figure 4A). Internal validation also revealed a similar result (Figure 4B).

**Figure 5:**
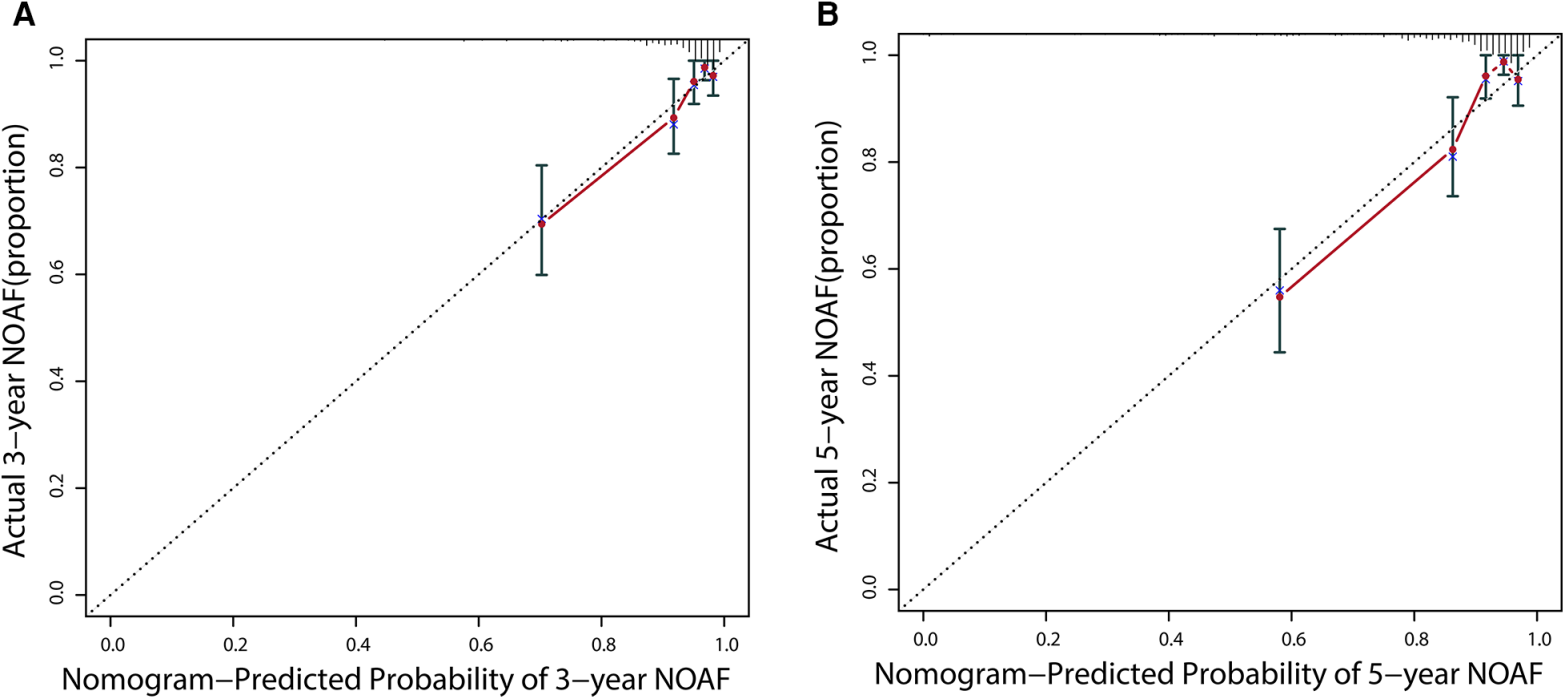
Calibration curves for 3- **(A)** and 5-year NOAF **(B)** of nomogram predictions.

Additionally, calibration would estimate the prediction–observation deviations depicted by the calibration plot. Figure 5 demonstrates the nomogram's excellent calibration for the 3- and 5-year NOAF predictions. A bootstrap resampling was also used to rectify the assessment above.

**Figure 6:**
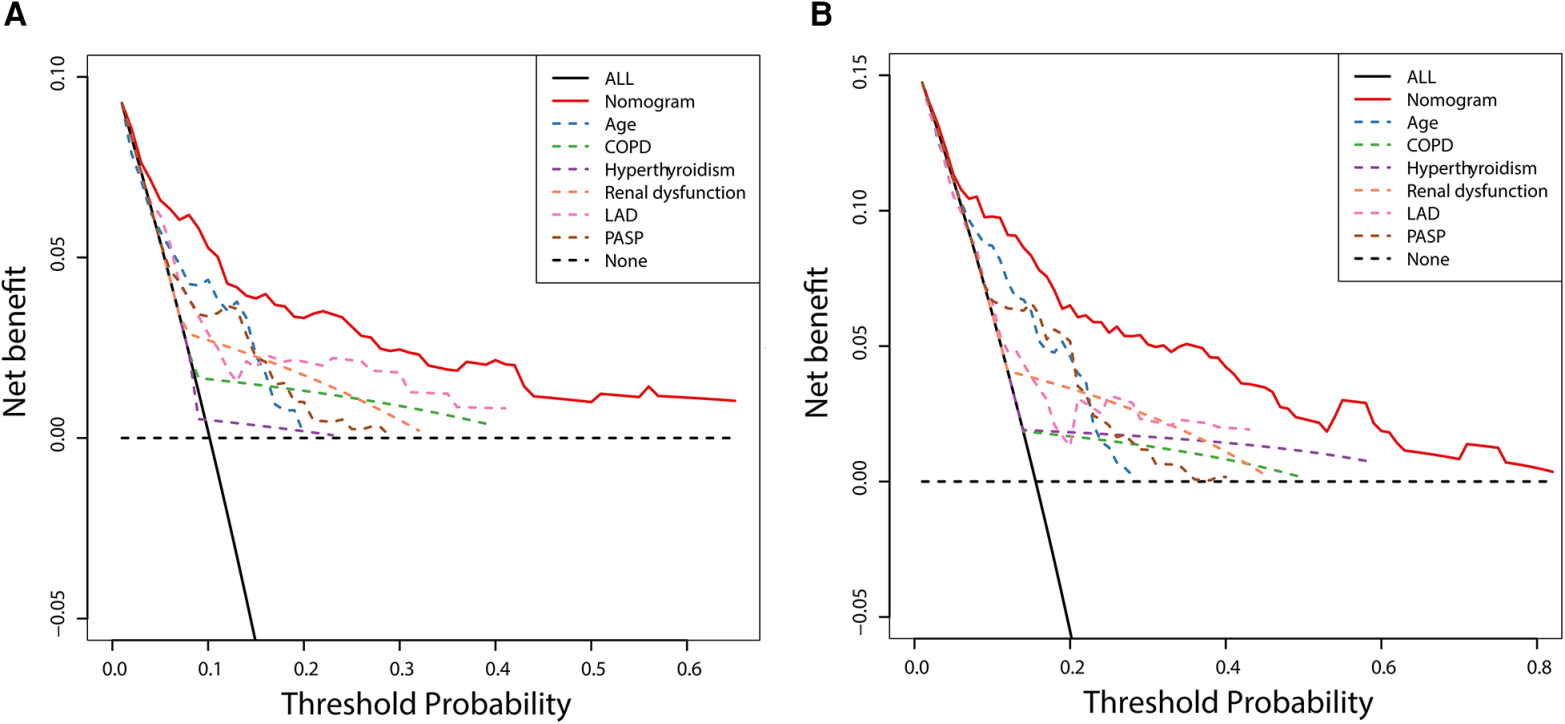
Decision curve analysis of nomogram compared with any single prognostic marker for 3- **(A)** and 5-year NOAF **(B)**.

The DCA was used to assess the clinical decision utility and net benefit. Figure 6 depicts that our nomogram could predict survival better than any single prognostic marker. Furthermore, the DCA instructed the nomogram's significant net clinical benefit. Overall, the nomogram demonstrated a strong ability to predict the prevalence of NOAF in HFpEF patients and a valuable clinical application.

### 3.4 Risk stratification based on the nomogram

**Figure 7:**
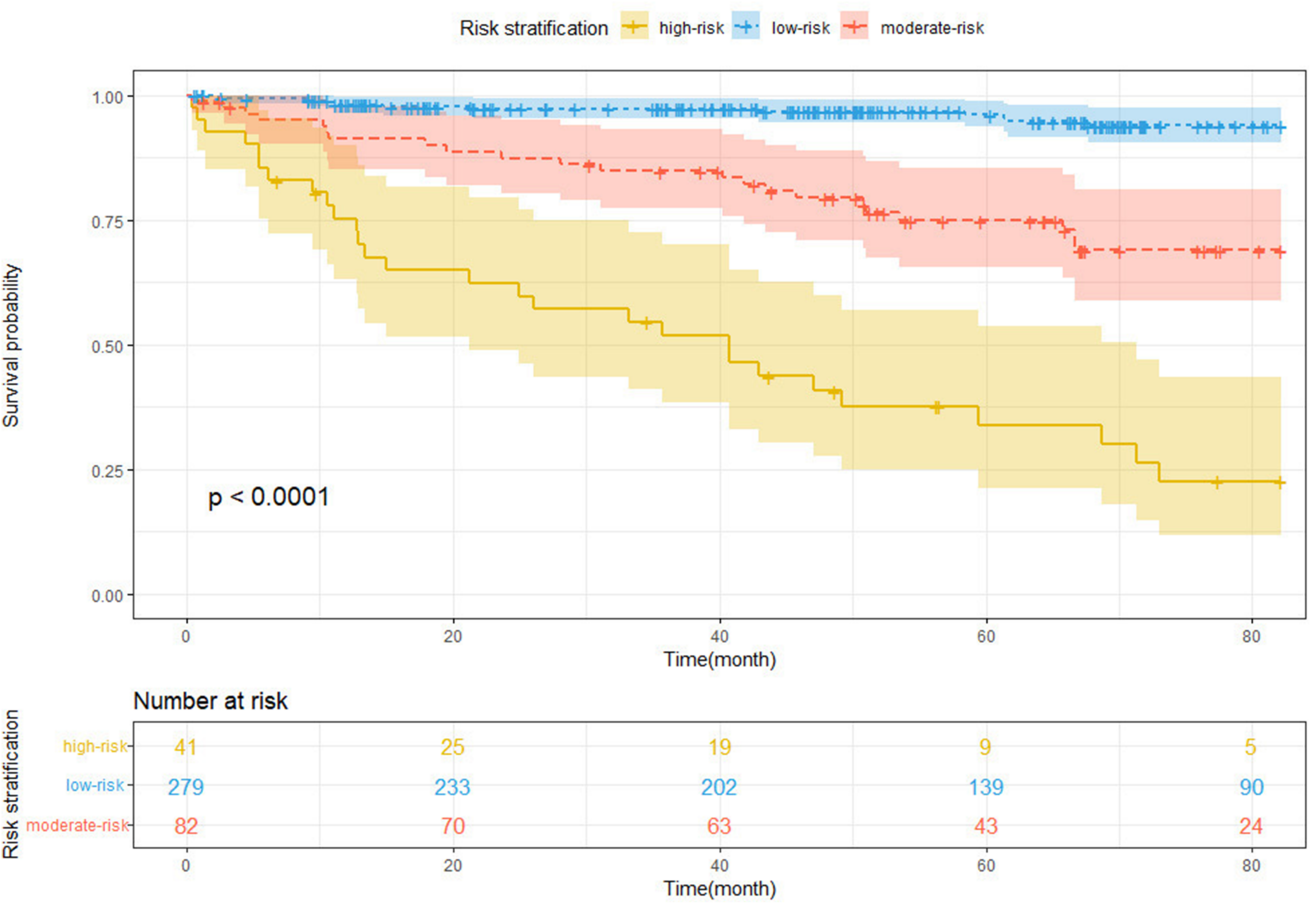
Kaplan–Meier curve for NOAF based on the prediction of nomogram.

For total points across all subjects, the X-tile software identified two ideal cutoff values, that is, 1.60 and 5.02, separating patients into the low-, moderate-, and high-risk groups. The risk stratification was evaluated using the Kaplan–Meier curves with a log-rank test (Figure 7, log-rank *P* < 0.0001). In comparison to the low-risk group, the moderate- and high-risk groups had significantly higher NOAF stakes [HR for the moderate-risk group: 6.17, 95% CI (3.05–12.46), *P* < 0.001; HR for the high-risk group: 22.91, 95% CI (11.62–45.17), *P* < 0.001].

### 3.5 Nomogram vs. mC_2_HEST score

The NRI and the IDI were used to assess risk prediction models' reclassification and discrimination performance. To test whether the “APART” nomogram and/or the addition of echocardiographic indicators improved the clinical risk classification across categories among the HFpEF patients, the continuous NRI and the IDI were calculated for the “APART” nomogram and mC_2_HEST score (Table 3). The nomogram demonstrated considerably positive NRI and IDI compared to the mC_2_HEST score both in the 3- and 5-year NOAF.

**Table 3:** Overall evaluation of the six-factor nomogram and the mC_2_HEST score.

Six-factor nomogram vs. mC_2_HEST score	NRI (95% CI)	*P*-value	IDI (95% CI)	*P*-value
3-year	0.356 (0.075–0.517)	0.008	0.110 (0.035–0.204)	0.004
5-year	0.239 (0.005–0.396)	0.036	0.086 (0.024–0.158)	<0.001

## 4 Discussion

A NOAF risk prediction model for HFpEF patients has not yet been established. Aiming at this group, echocardiographic indices were added to construct a six-factor nomogram. The nomogram was demonstrated, verified, evaluated simultaneously, and compared with the existing mC_2_HEST score. The “APART” nomogram further improved prediction accuracy and discrimination ability.

According to the multivariable analysis, the following were risk factors for incident AF in HFpEF patients: age, COPD, hyperthyroidism, renal dysfunction, LAD, and PASP. Prior studies also demonstrated that all these risk factors were associated with an elevated risk of AF.

In addition, we discovered an interesting phenomenon: the female sex tends to show a lower risk of AF in previously conducted research, including the mC_2_HEST score, while the male sex frequently has been linked to an increased risk of AF (13, 27, 28). Sex hormones may contribute to differences in outcomes. Based on previous experimental studies, estrogen may attenuate atrial electrical remodeling, decreasing susceptibility to AF (29). However, in our current study, female HFpEF patients did not have a lower risk of developing AF compared with males (Table 2, *P* = 0.714) possibly because most of the HFpEF patients we included were middle-aged or elderly (63.73 ± 10.18 years) and most of the females were perimenopausal or menopausal. The estrogen secretion had been dramatically reduced, diminishing the protective effect in females and reducing the risk of AF among different sexes. However, according to the CHA_2_DS_2_-VASc score for predicting stroke in patients with AF, the stroke risk of female patients with AF was higher than that of males under the same conditions. Hence, middle-aged and elderly females need extra attention for their atrial fibrillation and adverse events.

Given the significant relationship between atrial fibrillation (AF) and coronary artery disease (CAD), we conducted further analysis specifically on the CAD subgroup. We observed that the most prevalent clinical manifestation of CAD was NSTEMI-UA in both the non-NOAF and NOAF groups. Compared to the non-NOAF group, the NOAF group exhibited a lower incidence of STEMI and less history of PCI/CABG. A retrospective cohort study previously reported that AF patients had a lower prevalence of multivessel coronary artery disease (≥3 diseased vessels), less severe CAD obstruction, and primarily presented with NSTEMI-UA (30). The authors attributed this to AF potentially leading to reduced coronary oxygen supply or increased oxygen demand, thereby predisposing patients to NSTEMI. Furthermore, AF patients tend to receive more ACEI/ARB and oral anticoagulants and more comprehensive clinical and instrumental follow-up. These factors may collectively contribute to a less severe CAD presentation. Upon analyzing our data, we identified similar trends. However, our study initially encompassed patients without AF, thus excluding the aforementioned AF-related mechanisms. It appears that the occurrence of adverse outcomes was more closely tied to the subsequent treatment choices. Specifically, more severe STEMI cases were often managed with myocardial reperfusion therapy, whereas milder NSTEMI-UA cases were treated conservatively with medication. It is plausible that myocardial reperfusion therapy could potentially reduce the incidence of AF in CAD patients. Future research in this area is warranted.

Secondary analyses of the Framingham Heart Study (FHS) suggested that echocardiography might be valuable for reclassifying the risk for individuals with valvular heart disease or HF (13). This was confirmed in our current study. Among the common echocardiographic indices, LAD and PASP are most closely related to AF, and their higher values are more likely to predict AF, consistent with previous studies (31, 32). There are also several biomarker-based scoring systems for predicting AF events. These biomarkers are not easy to obtain and fluctuate dynamically, so it may not be appropriate to use biomarkers to predict the results after several years at baseline. Hence, these biomarker variables were not included in this study.

In examining whether the addition of the echocardiographic indices further improves the model performance based on the mC_2_HEST score, novel metrics IDI and RNI are introduced as alternatives to the increase in the area under the ROC curve. The reasons are as follows: first, the AUC depends strongly on the baseline model, which is true to a lesser degree for the integrated discrimination improvement. In contrast, this phenomenon is much weaker for the IDI (33). Second, the continuous version of the NRI depends mainly on the effect size of the added predictor, which is more suitable for evaluating the improvement effect of new indices on the model (34, 35). This will make it easier to understand the value that echocardiographic indicators bring to the current study. The RNI values of the 3- and 5-year NOAF are 35.6% and 23.9%, respectively, indicating that the APART nomogram improves the correct classification by 35.6% and 23.9%, respectively, compared with the mC_2_HEST score.

The comorbidities of advanced age, hyperthyroidism, renal dysfunction, and COPD predispose HFpEF patients to new-onset atrial fibrillation. In addition, while we pay attention to the changes in ejection fraction (EF%), we also need to be concerned about the indices of LAD and PASP in echocardiography. The changes in these indices will be closely related to the AF and patient prognosis. In patients who are at high risk for developing AF, aggressive screening could be advantageous. In patients with low risk, AF screening may be skipped.

There are several limitations in this study. This was a single-center retrospective study with limited representativeness. Moreover, we note that AF is not occasionally asymptomatic or paroxysmal, doubtlessly lacking in our cohorts. Our prediction of NOAF type is non-specific.

## 5 Conclusion

We have developed and validated the “APART” nomogram, including echocardiographic indices, to assess the individual risk of NOAF. The nomogram performed well in discrimination, calibration, clinical utility, and separative efficacy. The nomogram may bring clinical benefit to HFpEF patients.

## Data Availability

The original contributions presented in the study are included in the article/Supplementary Material; further inquiries can be directed to the corresponding author.
